# Supplementary material for: Vacuolar Proton Pyrophosphatase Is Required for High Magnesium Tolerance in Arabidopsis
Source: Int J Mol Sci. 2018 Nov 16;19(11):3617. doi: 10.3390/ijms19113617 (PMC6274811; doi:10.3390/ijms19113617)
Supplement: Supplementary file 1 [file ijms-19-03617-s001.zip › ijms-386020-AVP1-supplementary/ijms-386020-supplementary.pdf]

Supplementary Materials

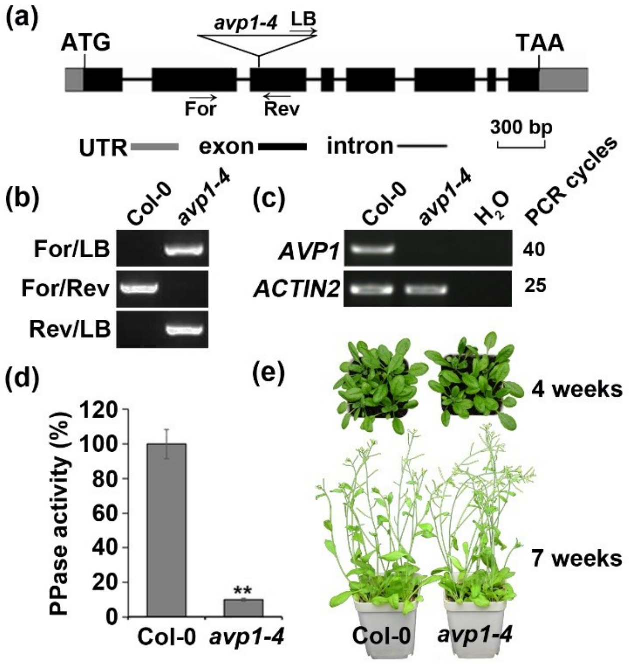

Figure S1. Molecular identification of *avp1-4* mutant. (a) Schematic diagram of T-DNA insertion in *avp1-4*. (b) PCR analysis to identify homozygous *avp1* knockout mutant. (c) RT-PCR analysis of *AVP1* gene expression in Col-0 and *avp1-4* knockout mutant. Expression of *ACTIN2* was analyzed as internal controls. (d) H^+-PPase hydrolytic activity of *avp1-4*. Results are shown as percentage of the Col-0 control activity. Values are mean ± SD of three replicate experiments. (e) Growth phenotype of *avp1-4*. Wild-type (Col-0) and *avp1-4* plants were transplanted to soil and grew under 12-h light/12-h dark cycles and pictures were taken 4-week and 7-week after transfer.

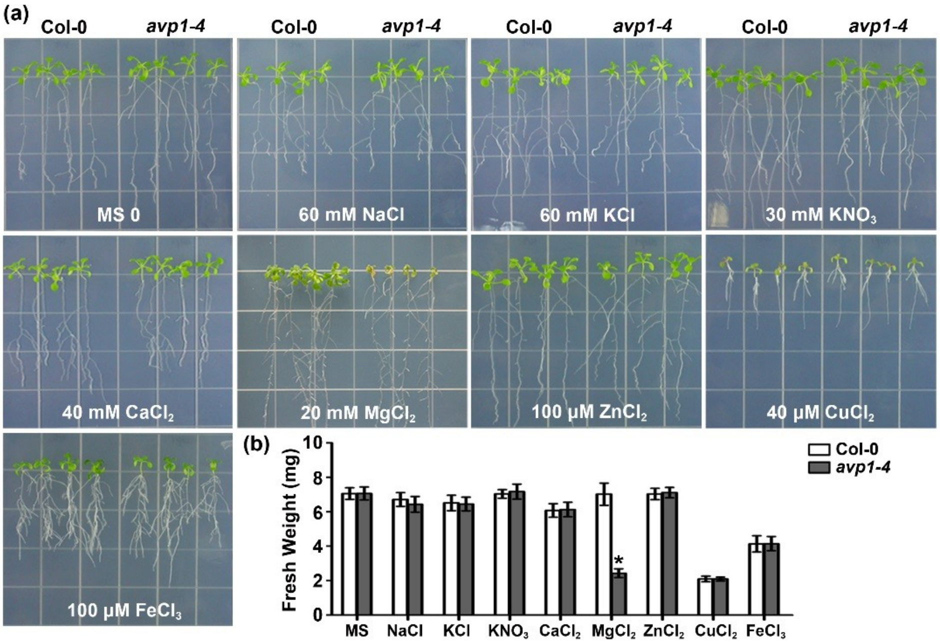

Figure S2. The *avp1-4* mutant is specifically sensitive to Mg^{2+}. Growth of wild-type Col-0 and *avp1-4* mutant plants under various ionic stress conditions. Five-day old Col-0 and *avp1-4* seedlings were transferred onto MS medium or MS medium supplemented with 60 mM NaCl, 60 mM KCl, 30 mM KNO_3, 40 mM $CaCl_2$, 20 mM $MgCl_2$, 100 μM $ZnCl_2$, 40 μM $CuCl_2$ or 100 μM $FeCl_3$, respectively. Pictures were taken on the 7th day after transfer. (b) Fresh weight of seedlings on the 7th day after transfer. Data are presented as mean ± SD of four replicate experiments. Asterisks indicate statistically significant differences compared with the Col-0 (Student's *t*-test, $^*P<0.05$).

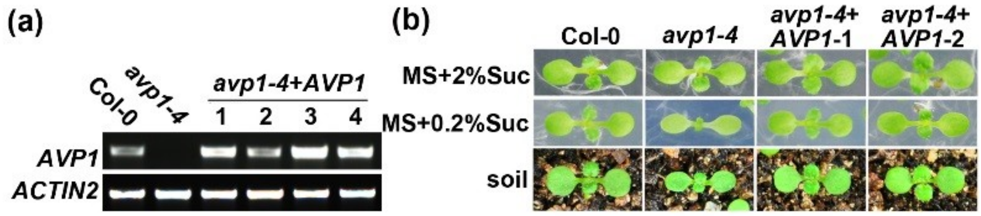

Figure S3. Functional complementation of *avp1-4*. (a) RT-PCR analysis of *AVP1* expression in Col-0, *avp1-4* and four randomly selected AVP1 complimentary

lines. (b) Morphological phenotypes of *avp1-4* mutant on different growth media. Col-0 and *avp1-4* mutant seedlings were grown for 8 days either on soil (bottom panel) or standard MS medium supplemented with 2% or 0.2% sucrose (top and middle panels).

Table S1. Primers Used in This Study.

Purpose	Name	Primer sequence (5′ to 3′)
avp1-4 mutant identification and RT-PCR	LP-AVP1	ACGACACCACCAGAACCTGCAAG
	RP-AVP1	ATGAGCAATGGGTAGCACATGGC
mgt6 mutant identification	LP-MGT6	ACTGGATGGAATGCGGAACAAG
	RP-MGT6	CCAAATCAAATCAACCCATAAAC
T-DNA left border primers	GKL	ATATTGACCATCATACTCATTGC
	LBa1	TGGTTCACGTAGTGGGCCATCG
MGT6 RT-PCR	MGT6RT-F	ACTGGATGGAATGCGGAACAAG
	MGT6RT-R	GGCGTAACCAAGAGTGACCATGA
CBL2 RT-PCR	CBL2RT-F	GCTCGTGCTCTCTCCGTCTTTC
	CBL2RT-R	GCCGCTGCTTGCTTTTGCTTTTG
CBL3 RT-PCR	CBL3RT-F	CTGAGTCCGGCATGAACCTGTC
	CBL3RT-R	TTCCCAAATTGTCTCCTCTGCTAA
ACTIN2 RT-PCR	ACT2RT-F	GGAAGGATCTGTACGGTAAC
	ACT2RT-R	GGACCTGCCTCATCATACT
