# Supplementary figures and images for: Vacuolar Proton Pyrophosphatase Is Required for High Magnesium Tolerance in Arabidopsis
Source: Int J Mol Sci. 2018 Nov 16;19(11):3617. doi: 10.3390/ijms19113617 (PMC6274811; doi:10.3390/ijms19113617)

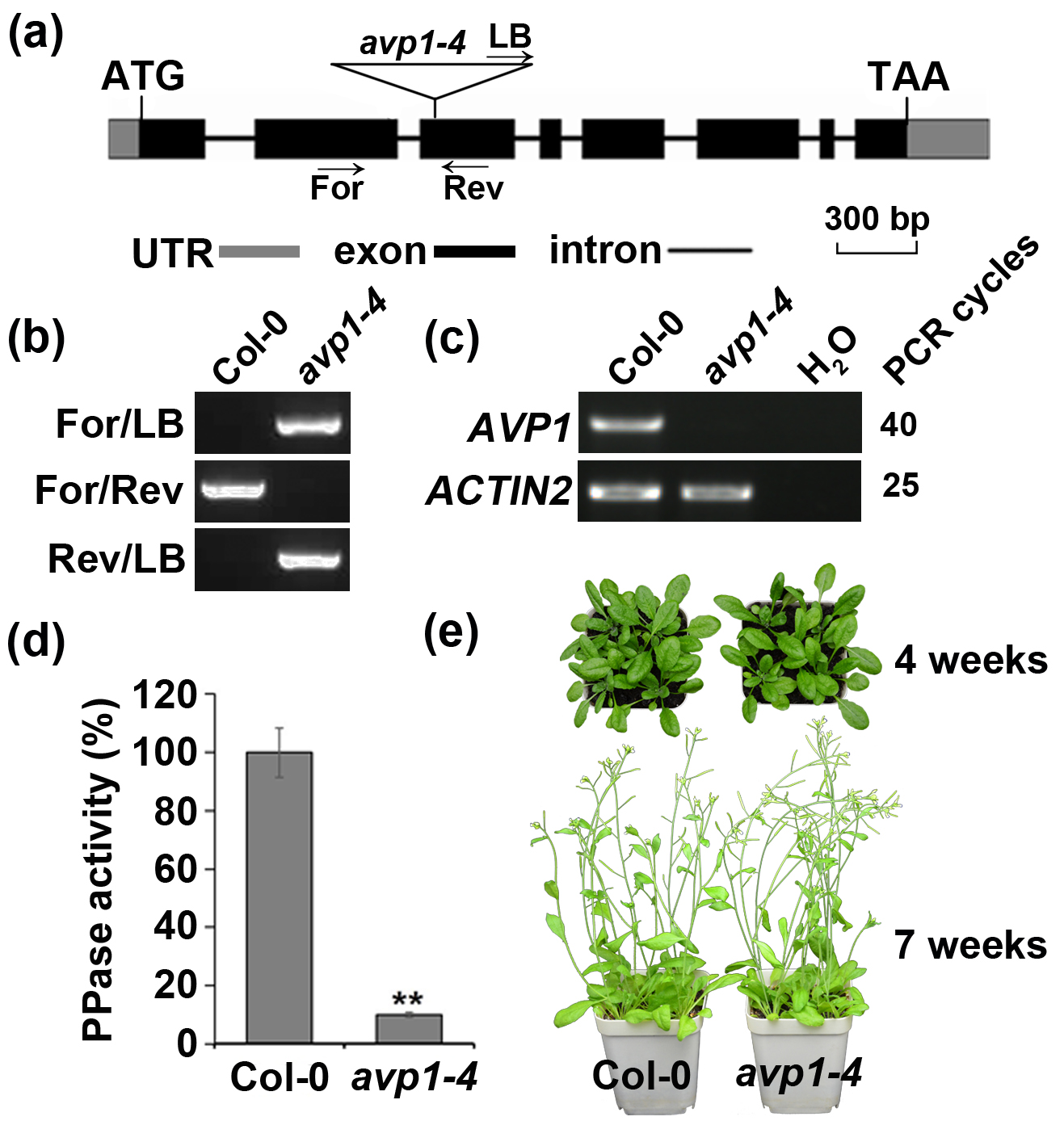

Supplement: Supplementary file 1 [file ijms-19-03617-s001.zip › ijms-386020-AVP1-supplementary/Figure S1.jpg]

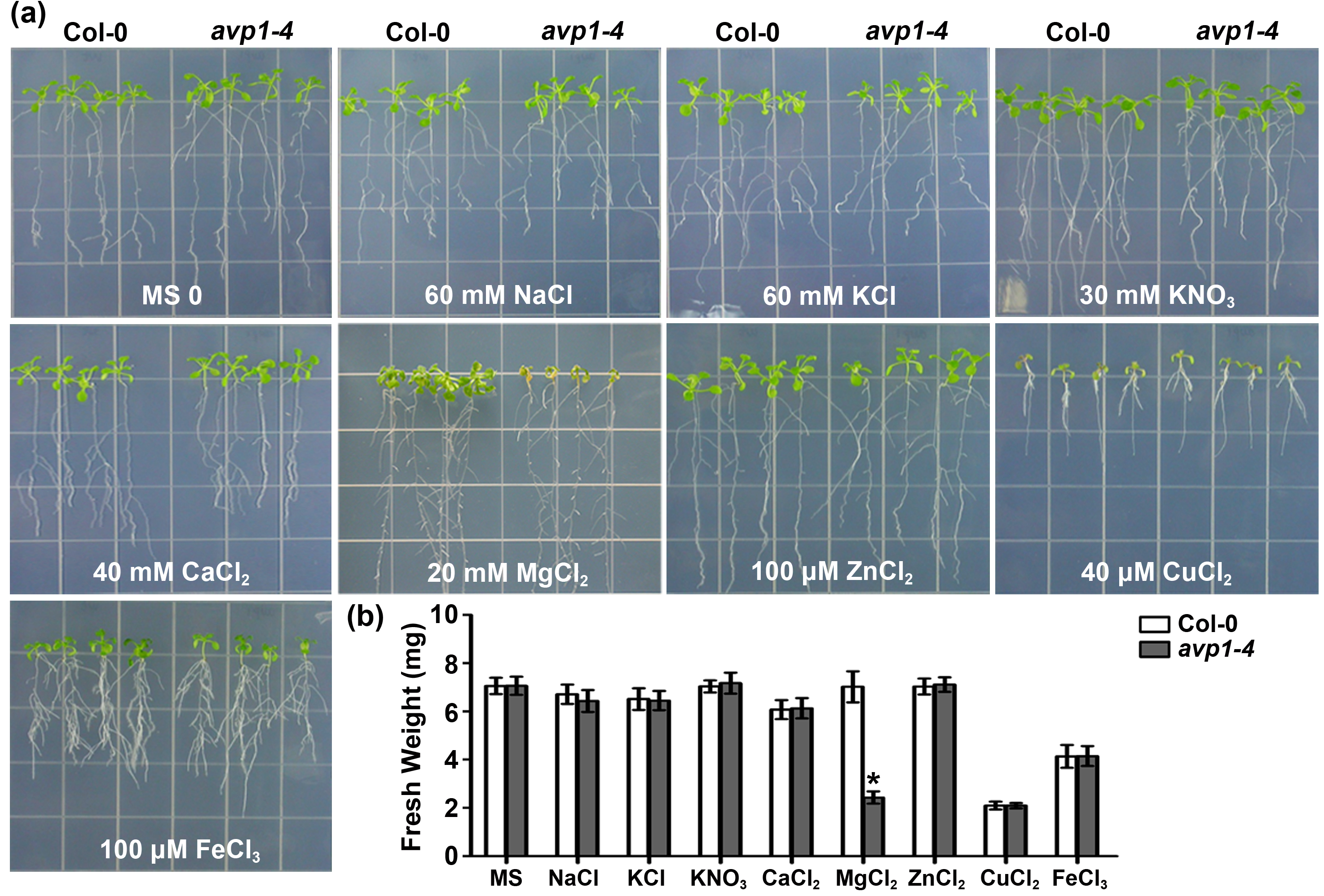

Supplement: Supplementary file 1 [file ijms-19-03617-s001.zip › ijms-386020-AVP1-supplementary/Figure S2.jpg]

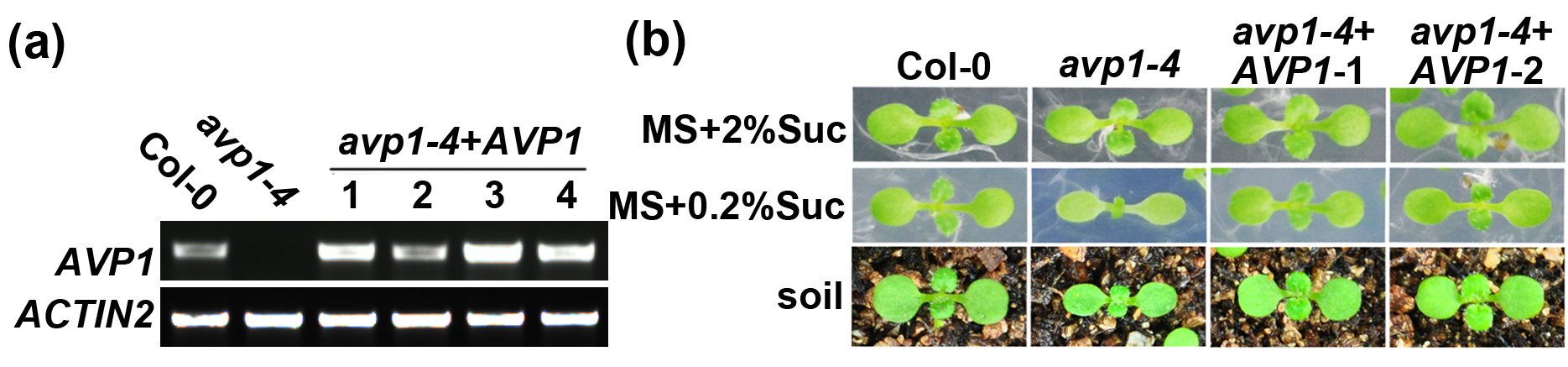

Supplement: Supplementary file 1 [file ijms-19-03617-s001.zip › ijms-386020-AVP1-supplementary/Figure S3.jpg]
